# Indoor Air Design Parameters of Air Conditioners for Mold-Prevention and Antibacterial in Island Residential Buildings

**DOI:** 10.3390/ijerph17197316

**Published:** 2020-10-07

**Authors:** Xueyan Zhang, Jingyi Liang, Beibei Wang, Yang Lv, Jingchao Xie

**Affiliations:** 1 School of Civil Engineering, Dalian University of Technology, Dalian 116024, China; xueyan@dlut.edu.cn (X.Z.); liangjingyi1216@163.com (J.L.); 17863900970@163.com (B.W.); 2 School of Construction Engineering, Beijing University of Technology, Beijing 100000, China; xiejc@bjut.edu.cn

**Keywords:** island residential buildings, dominant fungi, design parameters of air conditioner, mold-prevention and antibacterial

## Abstract

The climate characteristics of the islands in the Nansha Islands of China are a typical marine climate including high temperature, high relative humidity, high salt content, strong solar radiation, and long sunshine. These can provide suitable conditions for mold reproduction on the surface of the wall in a building. Therefore, mildew pollution on the wall for a long time can easily damage the building’s structure. It does not only directly affect the appearance of the building, but also indirectly affects the indoor environment and human health. In this paper, dominant fungi in the residential buildings on thee Nansha Islands of China are *Aspergillus*, *Penicillium,* and *Cladosporium*. Critical lines of temperature and relative humidity for mould growth on the interior surfaces of island residential building envelopes have been given and discussed. The results show that the risk of mould growth on the wall with different materials, from low to high, is reinforced concrete, aerated concrete block, coral aggregate, brick, and wood. Furthermore, in order to prevent the room regulated by air conditioner from being contaminated by mould, indoor air temperature should be set variable and controlled between 26 °C and 28 °C, the relative humidity should be changed between 50% and 80%.

## 1. Introduction

Several hundred species of fungal and bacterial can usually be found in indoor environments [1,2]. Moisture accumulation can lead to mold growth on the surface of building envelopes. Mold spores are generated inside a room and on the surface of the walls of different building materials, and can be transferred by the flowing air and the activities of personnel. However, spores can germinate and produce mycelium in a dormant state, and then reproduce under appropriate conditions. On one hand, the structure of building envelopes can be damaged by mold growth, which could cause a dark color or obvious color change on the surface of the wall. However, there is no obvious pigmentation when effective mold grows and it is not easily found, even if the number of molds is very rich. On the other hand, existing studies have shown that respiratory tract infections, asthma, dermatitis and other allergies, and even infectious diseases can be caused by exposure to or the inhalation of mold and its metabolites for a long time, as confirmed by the Institute of Medicine (IOM) and the World Health Organization (WHO) [3,4,5]. In Northern Europe and North America, according to the estimation, 20% to 40% of buildings are contaminated by indoor mold [6], which has a significant impact socially and economically [7,8]. For example, costs related to indoor mold pollution have been estimated by the United States and Scandinavia, where the results show that annual social and economic costs caused by high humidity and mold growth are 2.3–4.7 billion US dollars for allergic rhinitis and 1.1–2.3 billion US dollars for acute rhinitis [5]. Furthermore, the municipal annual costs for repairing public property damaged by high humidity and mold growth reached 5 billion euro, accounting for 1.9% of Finland’s GDP in 2012, and the annual cost of health problems caused by severe dampness and mold damage reached 450 million euro [9]. When mold grows on the wall, it is almost impossible to eradicate [10] as mold spores always exist in the air, whose content is directly related to the seasonal changes and specific conditions of outdoor climate [11]. The reproduction ability of mold spores is extremely strong, which can germinate and produce hyphae under a suitable condition. Therefore, the environmental parameters should be controlled before mold can germinate [12]. The affecting environmental parameters include temperature [13], relative humidity [14,15,16], nutrients [17], pH value [18], surface roughness, etc. [19]. Among them, temperature and relative humidity are the main factors impact on mold growth [20,21,22]. Therefore, indoor air temperature and relative humidity should be controlled before spores are germinated.

**Figure 1:**
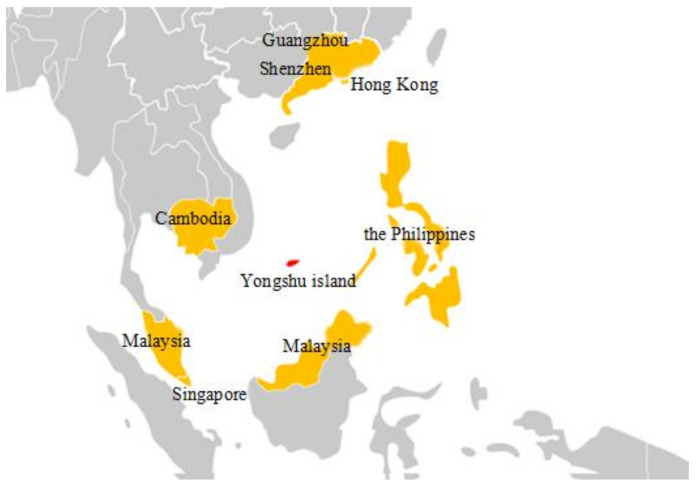
Countries and regions near the Nansha Islands, China.

The Nansha Islands are located in the south of China, and the geographical location is shown in Figure 1. Due to the typical Tropical Ocean monsoon climate and significant meteorological characteristics of high temperature and relative humidity, mold can grow on the surface of the wall effectively. In this paper, the dominant species of indoor mold in island residential buildings was determined by literature searches. The critical temperature and humidity for mold growth on the surface were found for when different materials were utilized on the building envelopes of residential buildings on Nansha Island. Finally, the indoor air design parameters and selection criteria of air conditioner for residential buildings on the Nansha Islands, China are proposed to prevent mold and bacteria. These results can be proposed and fill in the gaps in the design code for the heating, ventilation, and air conditioning of civil buildings.

## 2. Materials and Methods

### 2.1. Investigations and Measurements

Mold growth is affected by many factors such as air temperature, relative humidity, pH value, and operation of the air conditioner. Furthermore, other important factors in indoor mold contamination include geographical location, meteorological conditions, the materials and structures of the building envelopes, the phenomenon of moisture, personnel activities on the utilization of air conditioners, etc. The above factors were included in a questionnaire (shown in Appendix A) for investigation. A total of 224 questionnaires were effective in the Nansha Islands, China. A total of 138 out of 224 were in multi-story residential buildings and 106 out of 224 were in single family dwellings in some villages.

Measurements of indoor air environment parameters included temperature, relative humidity, air velocity, and CO_2_ concentration. Indoor air testing parameters were monitored at the breathing zone height (1.1 m above the floor) according to the National Indoor Air Quality Standard (GB/T 18883-2002) [23]. Characteristics of the above instruments are shown in Table 1. These measured data were recorded every five minutes.

**Figure 2:**
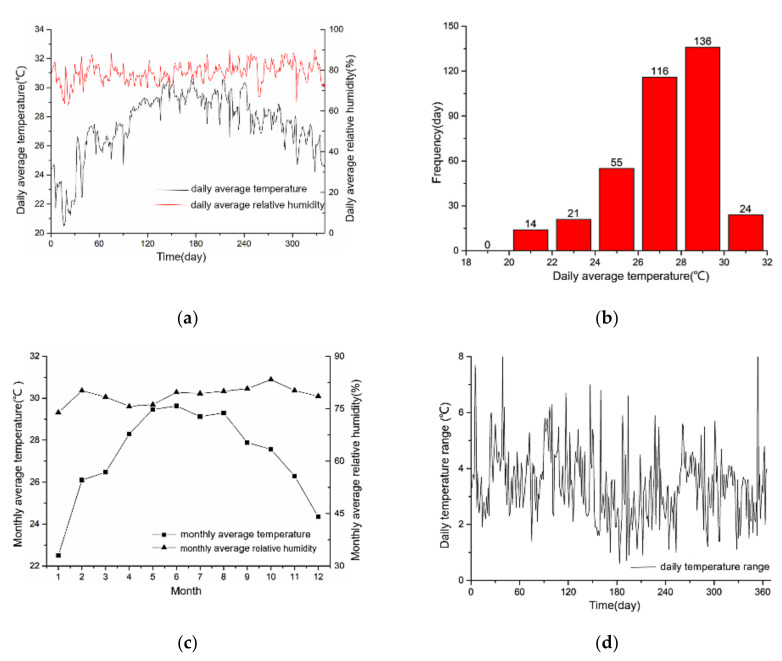

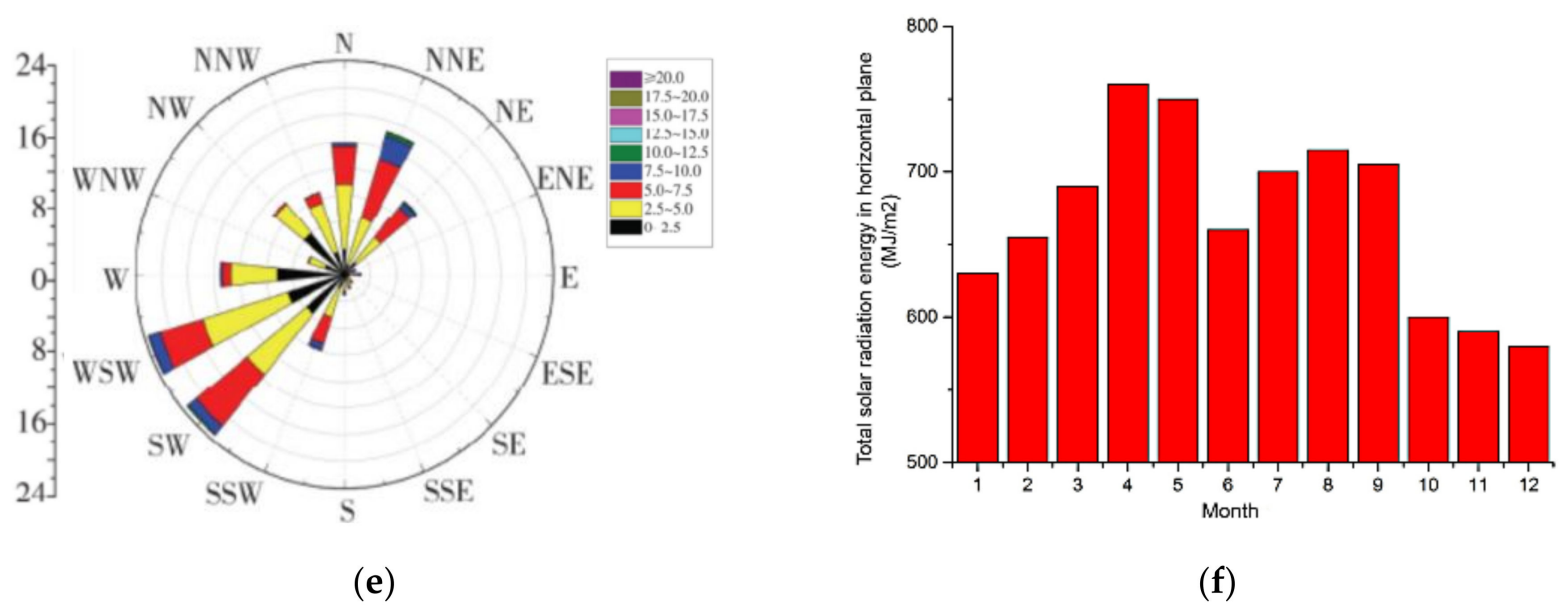
Climate analysis of Nansha Island. (**a**) Daily average temperature and relative humidity; (**b**) Frequency of daily average temperature; (**c**) Monthly average temperature and relative humidity; (**d**) Daily temperature variation; (**e**) Annual hourly wind rose; (**f**) Monthly solar radiation.

The characteristics of the outdoor environmental parameters in the Nansha Islands of China are shown in Figure 2. It can be seen that the daily average temperature exceeds 26 °C on 276 days, which accounts for 75.6% in a year. The lowest monthly average temperature is 22 °C in January, and the highest temperature is 29.6 °C in June. The annual temperature difference is 7.1 °C. The days with a temperature difference below five accounts for 90.1%.

Monthly average relative humidity of the whole year is above 75%. The frequency of wind speed higher than 5 m/s accounts for 31%. The daily solar radiation on the horizontal plane is more than 4.5 (kw·h)/m^2^. Above all, the characteristics of outdoor climate are high temperature, high relative humidity, high salt content, strong solar radiation, and long sunshine time. In addition, typhoon and heavy rainfall always occur in the monsoon period.

### 2.2. Materials of Building Envelopes

**Figure 3:**
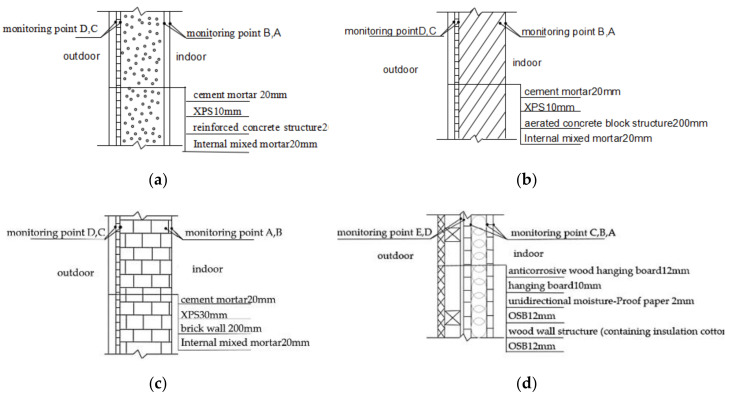

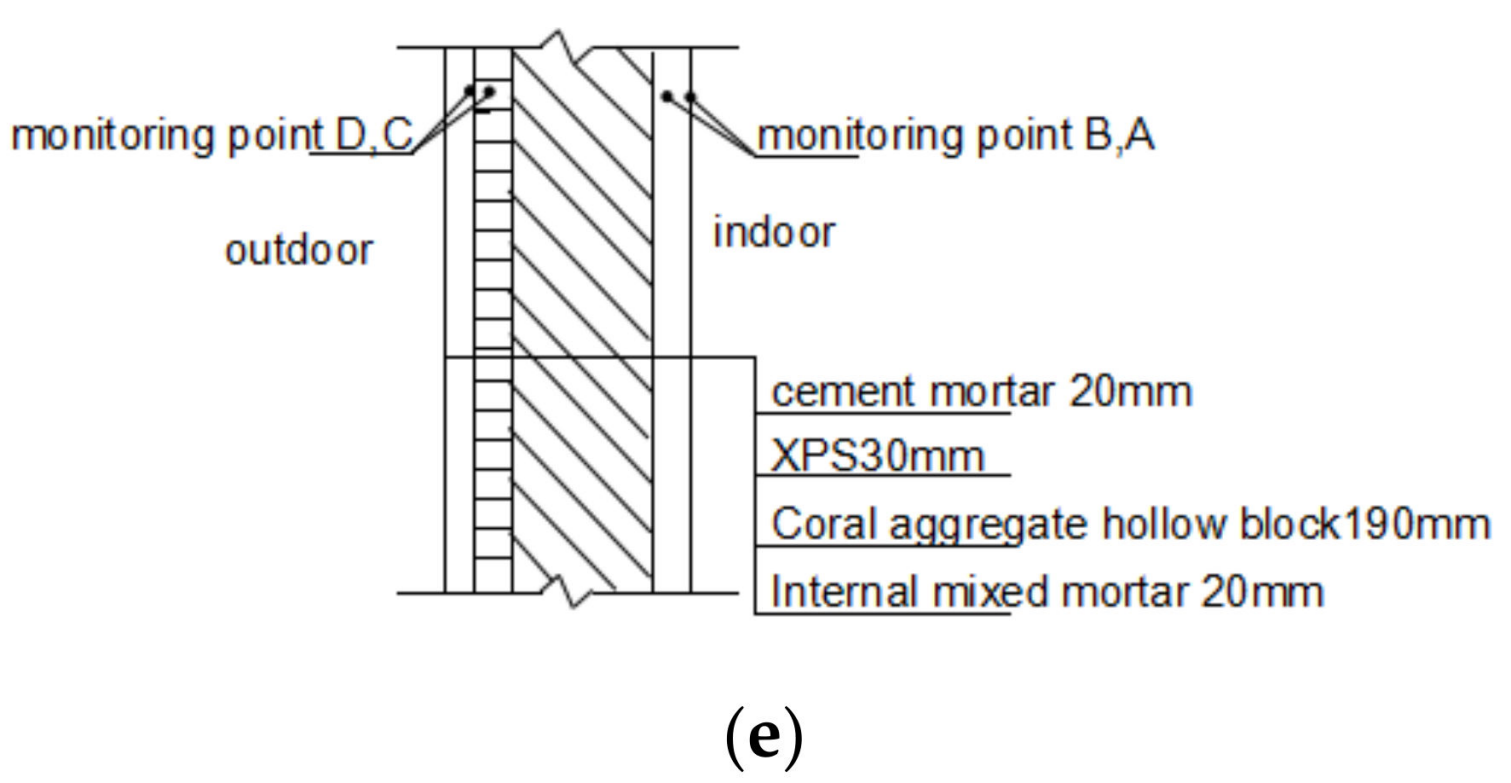
Different structures of building envelopes. (**a**) Reinforced concrete; (**b**) Aerated concrete block; (**c**) Brick wall; (**d**) Wood wall; (**e**) Coral aggregate wall.

According to the results of the investigation, the materials of building envelopes were reinforced concrete, aerated concrete block, brick and concrete, and wood in the Nansha Islands residential buildings of China. The moldy phenomenon of different types of walls is very serious. The mold growth risk on the surface of the wall from high to low was: wood wall, brick concrete, aerated concrete block, reinforced concrete, and coral aggregate wall [24,25,26,27,28]. According to the survey results, the structures of these five building envelopes and the layout of testing points are shown in Figure 3. The physical parameters of each material layer are listed in Table 2.

### 2.3. Use of Air Conditioner

The survey results showed that the indoor air temperature of the room with the air conditioner was between 22 °C and 28 °C, mainly concentrated at 25 °C. The operation period of air conditioners is from April to October. During other times, the main method is natural ventilation. A total of 86.1% of users preferred to turn off their air conditioners and open windows for ventilation at night, however, most people preferred to use air conditioners during the daytime.

### 2.4. Dominant Fungi

Among these 244 valid questionnaires, serious mold contamination accounted for 203 (83.2%), and the time of mold contamination was mainly from April to October. Asthma is one of the most common diseases, which accounts for 3.0%. In addition, respiratory diseases such as rhinitis, pharyngitis, symptoms of nasal and throat discomfort, and skin rash accounted for 52%. However, the results of other 41 questionnaires reflected that while there was no mold contamination in the buildings, 24 of them still reflected the above symptoms. These results show that there is a strong correlation between mold contamination and respiratory diseases.

Dominant fungi of indoor mold (on the surface or in the air) in island residential buildings on the Nansha Islands of China were investigated and the results are listed in Table 3, where the detection results of corresponding fungi are marked by “*√*”. Each detection rate percentage is given according to the reference [30,31,32,33,34,35]. According to these results, the dominant fungus in island residential buildings were *Aspergillus* and *Penicillium*, followed by *Cladosporium*. The growth trend of these fungus was isoline.

### 2.5. Numerical Modeling

#### 2.5.1. Heat and Moisture Transfer Model of the Wall

##### Model and Grid

The mathematical models used for simulation were based on the energy equilibrium and moisture equilibrium. The critical curve model of mold growth is the isopleth model [36]. In this paper, the computational domain was from the outside to the inside of the wall and included the inner surface of the wall. The method used for dividing the mesh was inner node. Compared to the outer node method, the nodes at the interface between layers contain only one material, so it is easy to discretize the mesh. In addition, the mass equation and energy equation can be discretized by the control volume method. The time format is implicit. As a result, the average absolute errors of temperature and relative humidity were 0.06 °C and 0.11%, respectively [35,36].

##### Assumptions

- Heat transfer and moisture transfer are a coupled process in the state of gas diffusion.
- Water molecules are attached on the wet side and fixed on the polymer, which is then transported through the expansion.
- Liquid water is transported in the porous materials of building envelopes.
- The hysteresis curve of moisture can be ignored.
- Under total pressure difference, the impact of air flowing and water freezing on the transport of enthalpy and moisture should be considered.

##### Boundary

Boundary conditions were considered and are listed in Table 4. In this study, the results of the simulation were for one year. The time step for calculation was set up one hour until the variation of temperature and relative humidity were stable.

##### Initial Conditions

**Figure 4:**
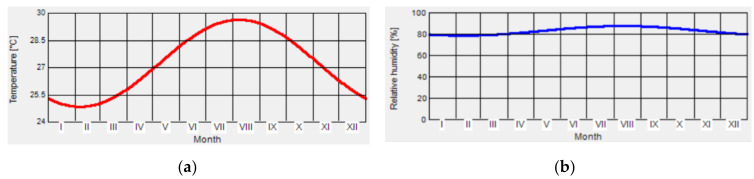
Indoor air temperature and relative humidity of a room under condition of natural ventilation. (**a**) Temperature; (**b**) Relative humidity.

The initial temperature on the surface of the wall was 20 °C, and moisture content on the surface of the wall was 80%. Annual variation of indoor air parameters are shown in Figure 4. The average temperature of indoor air in one year was 27.3 °C. Relative humidity of one year exceeded 80%.

##### Simulation Conditions

According to the results of the 244 questionnaire surveys, the period of air conditioner operation in one year was from April to October. Simulation conditions included: natural ventilation, air conditioner operating at night, air conditioner operating in the daytime, and air conditioner operating 24 h a day.

#### 2.5.2. Prediction Model of Mold Growth

Series of WUFI software was developed by the Fraunhofer Institute of Building Physics (IBP). WUFI-Bio, an additional software of the WUFI series software, was applied to estimate the risk of mold growth to the wall. The heat and moisture transfer process of the wall can be simulated by WUFI-Plus. Then, the calculated results by WUFI-Plus can be taken as the boundary condition and input into WUFI-Bio to simulate the mold growth process of the wall.

##### Calculation Process

**Figure 5:**
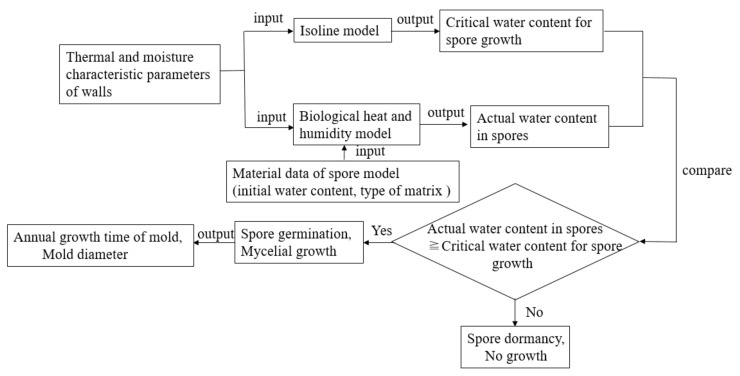
Calculation process via WUFI-Bio.

The calculation process of the WUFI-Bio software is shown in Figure 5.

##### Simplified Conditions

**Figure 6:**
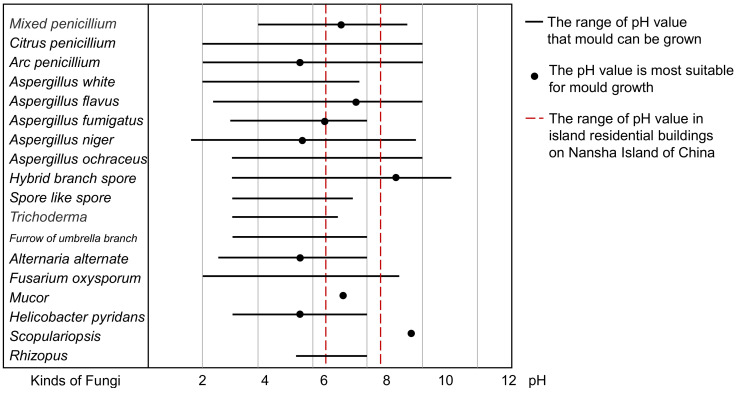
pH value for mold growth [39,40,41,42,43,44,45,46].

This model was used to simulate mold growth inside the wall or on the surface of the wall. The model of heat and moisture transfer can only be used to evaluate mold growth. The simplifications were as follows:

- According to the literature investigation, the spatial and temporal distribution of the pH value of the Nansha Islands’ marine climate showed that the general surface layer (0–10 m) on the building surface was 8.2–8.5 [37]. After sedimentation, the pH value was between 6.5 and 7.2 [38], which is neutral. Therefore, it was considered that the pH range in the salt fog environment of Nansha Islands of China was from 6.5 to 8.5. As shown in Figure 6, suitable pH values for mold growth are given [39,40,41,42,43,44,45,46]. It can be seen that *Penicillium* and *Aspergillus* are the most suitable for growth.
- Some factors should be considered such as illumination, oxygen content, and surface roughness. Most molds grow both in anaerobic and aerobic environments. Molds cannot synthesize organic matter by itself, so the condition of light cannot significantly affect mold growth [47,48,49].
- The influence of sediment or pollutants on the surface of building envelopes was not considered.
- The diffusion resistance of mold spores cannot be measured. Actually, the balance difference of water vapor inside the spore is very small under unsteady conditions. In addition, the value was as the same as the isoline model under steady conditions.

##### Assumptions

Through WUFI-Bio simulation calculation, the annual growth diameter variation of bacteria was influenced by the structures and materials of different walls, and the utilization behavior of the air conditioner, and the temperatures and relative humidity of indoor air can be obtained. Surface roughness of different building materials directly affects the performance of moisture storage, then indirectly affects mold growth [47]

## 3. Results

### 3.1. Annual Growth Diameter of Bacteria

#### 3.1.1. Natural ventilation

**Figure 7:**
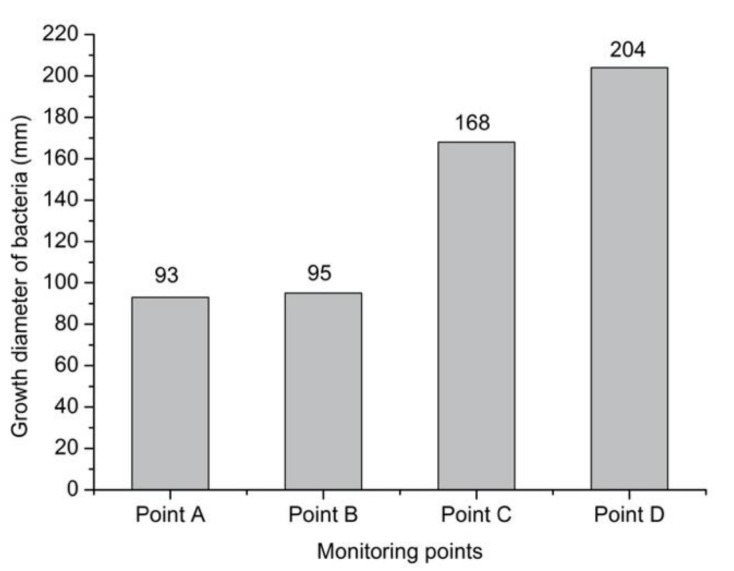
Annual growth diameter of bacteria at each monitoring point layer under the natural ventilation condition.

Under the condition of natural ventilation, the diameter variation of mold growth on each monitoring point of building envelope, constructed by reinforced concrete, is discussed. The results are shown in Figure 7, where the growth diameter of each monitoring point exceeds the limit value (50 mm one year), and the result at monitoring point D exceeded 200 mm a year. Furthermore, the risk of mold contamination exists at every monitoring point, and the risk from high to low was point D, point C, point B, and point A.

#### 3.1.2. Use of Air Conditioner in the Daytime

**Figure 8:**
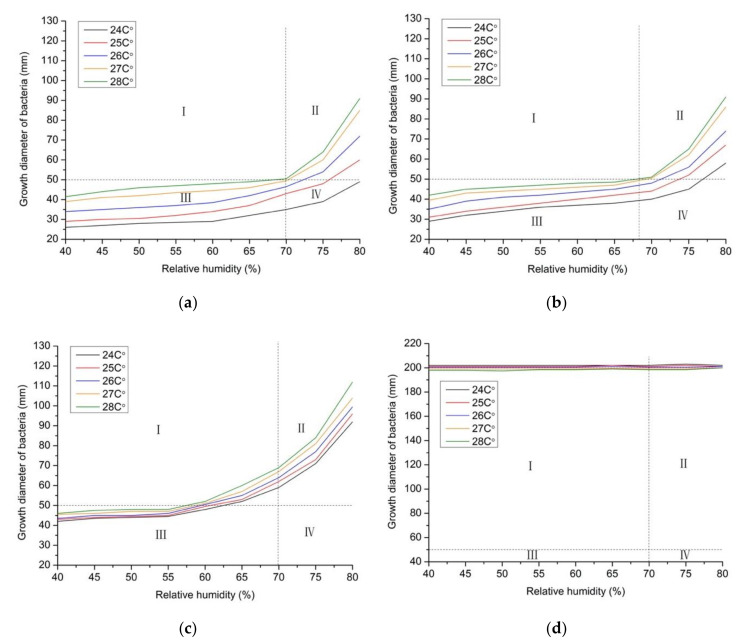
Annual growth diameter of bacteria at each monitoring point by using air conditioning in the daytime. (**a**) Point A; (**b**) Point B; (**c**) Point C; (**d**) Point D.

In Figure 8, Zone I refers to a thermal comfort area with the risk of mold contamination; Zone II refers to a thermal discomfort area with the risk of mold contamination; Zone III refers to a thermal comfort area without the risk of mold contamination; and Zone IV refers to a thermal discomfort area without the risk of mold contamination. The vertical dash line was 70% of the maximum relative humidity to meet the thermal comfort area, and the horizontal dash line was the critical value of 50 mm per year of growth diameter with mold contamination. Therefore, the graph was divided into four parts.

As shown in Figure 8, when the air conditioner was operated in the daytime, the annual growth diameter of bacteria at each monitoring point under different conditions of temperature and relative humidity are given. In addition, the risk of mold contamination was analyzed combined with the thermal comfort area. As the temperature variation was from 24 °C to 28 °C and the relative humidity variation was from 40% to 80%, the growth length of mycelium on each monitoring point was directly proportional to the indoor air temperature and relative humidity. Furthermore, the risk of mold contamination was always generated on monitoring point D, which was not affected by any indoor environmental factors. However, under the same temperature and relative humidity, the order of mycelium growth diameter from large to small was point D, point C, point B, and point A, and the order of risk from high to low was point D, point C, point B, and point A.

### 3.2. Critical Line of Temperature and Relative Humidity

**Figure 9:**
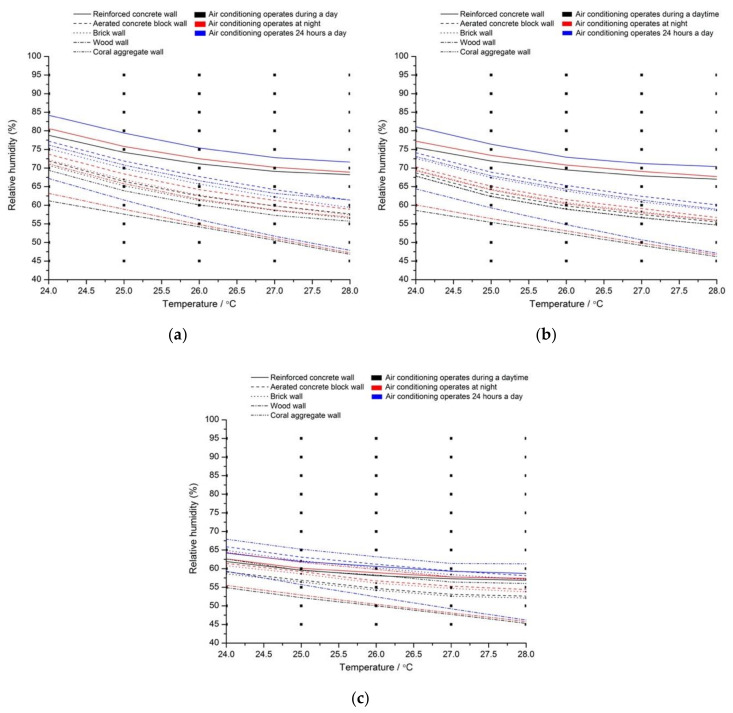
Critical lines of temperature and humidity of an air conditioner for mold prevention on the internal surface of wall. (**a**) Point A; (**b**) Point B; (**c**) Point C.

The annual growth diameter of mold is 50 mm, and the critical lines of temperature and relative humidity of each monitoring point under different conditions are given in Figure 9. Above the critical line, it illustrates that there is a risk of mold contamination in the room. In contrast, it illustrates that there is no risk of mold contamination in the room under the same indoor environment. By comparing these critical lines of five building materials, it can be seen that the indoor air temperature and relative humidity of air conditioners for preventing mold and bacteria in residential buildings on Nansha Islands can be given (Figure 9). By comparing three working conditions of the air conditioner, the risk of mold contamination in the room using the air conditioner in the daytime was the highest. The order of annual bacteria growth diameter from large to small was air conditioner operated in the daytime, at night, and 24 h a day. In addition, risk of mold growth on the wall, constructed with different five materials, from low to high was reinforced concrete, aerated concrete block, coral aggregate, brick, and wood.

Therefore, our suggestions are as follows. First, materials of the residential buildings should be reinforced concrete and aerated concrete block. Second, the air conditioner should be turned on all day, especially the dehumidification function. Third, the indoor air temperature should be kept at 26 °C, and the indoor air relative humidity should be maintained at 50% by the air conditioner.

## 4. Discussions

The occurrence of asthmatic symptoms is higher in most island residential buildings. The impacts of environmental parameters including temperature, relative humidity, nutrients, pH value, surface roughness, ventilation rate, etc., on mold growth have been researched in [50,51,52,53,54]. Temperature and relative humidity are the most important factors in the room regulated by an air conditioner. Fungi is more suitable for growth in the range of 15~40 °C [55]. Some investigations have studied the impact of temperature and relative humidity on the mold growth and reproduction in the building with an air conditioning system. It was found that a higher RH level is the main factor impacting on mold growth while the indoor air temperature was between 24 °C and 28 °C by air conditioner [56]. However, a reduction could be found in bacteria viability when temperatures were above 24 °C [57]. According to the design code for the design of heating, ventilation and air conditioner of civil buildings (GB 50736-2016) [58], the air design temperature and relative humidity only combine with the human comfort zone. Therefore, on the particularity of island buildings, the critical indoor air design temperature and relative humidity of the air conditioner to prevent mold and bacteria should be taken into careful consideration. In addition, results in this research are similar to results in [56,57]. Indoor air design temperature should be variable, and controlled between 24 °C and 28 °C, while relative humidity is between 40% and 80%.

## 5. Conclusions

Through literature reviews, field surveys, and numerical simulations, dominant species of mold in island residential buildings in Nansha Islands of China were obtained. The dominant species were *Aspergillus*, *Penicillium*, and *Cladosporium*.

Mold growth risk on the wall with different materials from low to high was reinforced concrete, aerated concrete block, coral aggregate, brick, and wood.

Indoor air design parameters for air conditioners for island residential buildings on the Nansha Islands of China were proposed to prevent mold and act as an antibacterial. The indoor air design temperature should be variable, and controlled between 26 °C and 28 °C, with the relative humidity between 50% and 80%.

The influence of local microclimate formed by wind speed, wind direction, rainfall, and other meteorological characteristics on mold growth needs to be further studied. The development of new envelope materials could also be further researched.

## Figures and Tables

**Table 1:** Characteristics of instruments for measuring indoor environmental parameters.

Indicator	Instrument	Rang	Accuracy
Temperature	WSZY-2 Recorder	−40~100 °C	±0.5 °C
Relative humidity	WSZY-2 Recorder	0~100% RH	±3%
Air velocity	WFWZY-1 Recorder	0.05~30 m/s	±0.05 m/s
CO_2_ concentration	WEZY-1S	0~5000 ppm	±50 ppm

**Table 2:** Each of the material property parameters of the above building envelopes [29].

Material	Density kg/m^3^	Porosity m^3^/m^3^	Specific Heat Capacity J/(kg·K)	Thermal Conductivity W/(m·K)
Cement mortar	2000	0.28	850	0.930
XPS	40	0.95	1500	0.030
Reinforced concrete	1600	0.31	850	1.740
Aerated concrete	600	0.72	850	0.140
Brick	1890	0.28	860	0.955
Oriented strand board	600	0.60	1400	0.120
Wood wall (containing fiber insulation cotton)	30	0.97	800	0.047
Unidirectional moisture-proof paper	1800	0.25	1600	0.177
Anticorrosive wood hanging board	470	0.52	2000	0.150
Coral aggregate hollow block	1151	0.31	6300	0.590
Internal mixed mortar	1780	0.28	850	0.700

**Table 3:** Dominant fungi in island residential buildings near Nansha Islands [30,31,32,33,34,35].

Kinds of Fungi	Countries and Regions					
	Guangzhou in China (Ratio %)	Shenzhen in China (Ratio %)	Hongkong in China (-)	Malaysia (-)	Cambodia (Ratio %)	Singapore (-)
*Asperaillus*	√ (30.2%)	√ (15.58%)	√	√	√ (76.3%)	√
*Penicillium*	√ (28.6%)	√ (29.7%)	√	√	√ (74.9%)	√
*Cladosporium*	√ (26.7%)	-	√	-	-	√
*Rhizopus*	√ (5.6%)	-	-	√	-	-
*Bipolar Fungi*	-	-	-	-	-	√
*Fusarium*	-	-	-	√	-	√
*Zygomycetes*	-	-	-	√	-	-
*Saccharomyces*	-	-	-	-	-	√

**Table 4:** Boundary conditions.

Equation	Heat and Humidity Transfer Mechanism	Expression of Boundary Conditions	Required Data	Input Data
Equilibrium Equation	Vapor diffusion	Vapor diffusion coefficient	Indoor and outdoor air temperature and relative humidity (steam partial pressure)	1. Typical meteorological year data (hourly temperature, relative humidity, solar radiation, rainfall, wind direction, wind speed, etc.). 2. Indoor design air parameters and air-conditioning use behavior. 3. Ventilation rate of air-conditioning is 0.5 times/h 4. Initial temperature was 20 °C and relative humidity was 80%.
	Rain	Rainwater model and applied flux	Rainwater flow density of horizontal plane, rainwater flow density perpendicular to the wall, outdoor wind direction and wind speed	
	Water film	Surface value and applied flux	Water pressure, flow rate, flow temperature, outdoor air temperature	
	Heat conduction	Heat conductivity coefficient	Indoor and outdoor air temperature and relative humidity	
Energy Equation	Solar short-wave radiation	Solar radiation model and applied flux	Direct solar radiation, scattered solar radiation, shielding	The outer surface absorption rate of short-wave radiation was set at 0.6, and the emissivity of long wave radiation was set at 0.9.
	Solar long wave radiation	Boltzmann calculation	Outdoor air temperature and relative humidity, cloud cover	Typical meteorological year data.

## Appendix A

Questionnaire on structure, humidity, and mold of island residential buildings on the Nansha Islands of China

**I. Personal information**

Gender: Age:

**II. Residential building information**

1. What is the built time of your building?

□ Before 1980 □ 1980~1990 □ 1991~2000 □ 2001~2010 □ Since 2011

2. What is the material of the building envelope?

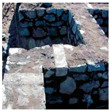
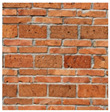
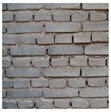
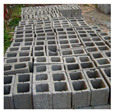

			
□ Rubble brick wall	□ Red brick wall	□ Dust brick wall	□ Hollow brick wall

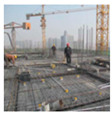
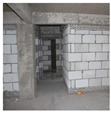
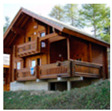
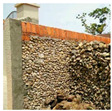
			
□ Reinforced concrete	□ Aerated concrete block	□ Wood	□ Coral Aggregate

3. What is the material on the inner surface of the wall?

□ Wooden board □ Lime mortar □ Latex paint □ Cement □ Wall paper □ Ceramic tile □ Other

4. How about the natural ventilation in your building? □ Best □ Better □ General □ Bad □ Worse

5. How is your building after ventilation? □ No obvious smell at all, fresh air □ No obvious smell, good air □ Peculiar smell, common air quality □ Obvious smell, need ventilation for a long time □ Other

**III. Moisture feeling and physical condition**

1. How do you feel during the day time? □ Very dry □ Dry □ Moderate □ Wet □ More wet

2. How do you feel at night? □ Very dry □ Dry □ Moderate □ Wet □ More wet

3. Have you ever suffered from asthma during your stay? □ Yes □ No

**IV. Moisture and mold conditions**

1. How about water leakage or water seepage in your building? (Single or multiple choice)

□ Water seepage on the roof □ Water seepage on the wall

□ Water seepage through doors and windows □ No water seepage or leakage

2. Where is the water leakage or water seepage in your building? (Single or multiple choice)

□ Floor □ Floor corner □ Ceiling □ Ceiling corner □ Wall □ Other □ No moisture

3. When does moisture regain or water droplets appear during one year? (Single or multiple choice)

□ January □ February □ March □ April □ May □ June □ July □ August

□ September □ October □ November □ December □ No resurgence

**V. Use of air conditioner**

1. What is the set temperature of air conditioner in your home?

□ ≤18 °C □ 19 °C □ 20 °C □ 21 °C □ 22 °C □ 23 °C □ 24 °C □ 25 °C □ 26 °C □ 27 °C □ ≥28 °C

2. When do you use air conditioner during a year? (Single or multiple choice)

□ January □ February □ March □ April □ May □ June □ July □ August

□ September □ October □ November □ December

3. When do you use air conditioner in a day? (Single or multiple choice)

□ 7 a.m.~9 a.m. □ 9 a.m.~11 a.m. □ 11 a.m.~1 p.m. □ 1 p.m.~3 p.m. □ 3 p.m.~5 p.m. □ 5 p.m.~7 p.m.

□ 7 p.m.~9 p.m. □ 9 p.m.~11 p.m. □ 11 p.m.~7 a.m. in the next day

4. When do you turn off the air conditioner and open the windows for ventilation during the year? (Single or multiple choice)

□ January □ February □ March □ April □ May □ June □ July □ August

□ September □ October □ November □ December

5. When do you turn off the air conditioner and open the windows for ventilation in a day? (Single or multiple choice)

□ 7 a.m.~9 a.m. □ 9 a.m.~11 a.m. □ 11 a.m.~1 p.m. □ 1 p.m.~3 p.m. □ 3 p.m.~5 p.m. □ 5 p.m.~7 p.m.

□ 7 p.m.~9 p.m. □ 9 p.m.~11 p.m. □ 11 p.m.~7 a.m. in the next day
